# Ultrasound Examination and Navigation for Repeat/Delayed Reconstruction of the Ankle Extensor Tendons

**DOI:** 10.3390/diagnostics11081408

**Published:** 2021-08-04

**Authors:** Kamal Mezian, Karolína Sobotová, David Zámečník, Levent Özçakar

**Affiliations:** 1 Department of Rehabilitation Medicine, First Faculty of Medicine, Charles University and General University Hospital, 128 00 Prague, Czech Republic; 2 Department of Rehabilitation and Sports Medicine, Second Faculty of Medicine, Charles University and University Hospital Motol, 150 06 Prague, Czech Republic; sobotovakarolina@gmail.com; 3 Department of General Surgery & Traumatology, Municipal Hospital, 412 01 Litoměřice, Czech Republic; davyzam@seznam.cz; 4 Department of Physical and Rehabilitation Medicine, Hacettepe University Medical School, Ankara 06100, Turkey; lozcakar@yahoo.com

**Keywords:** ultrasound, tibialis anterior, extensor digitorum longus, lower extremity, muscle, tendon

## Abstract

Herein, we describe a 46-year-old woman with persistent pain and weakness in her left ankle/foot one year after surgical repair of all three ankle extensor tendons following a penetrating injury. This report presents a unique case whereby US imaging played a paramount role in the diagnosis and surgical management of a previous nonanatomic repair of the ankle extensor tendons after a penetrating injury one year prior. The above-quoted findings were subsequently corrected with end-to-end sutures. On the third postoperative month follow-up, the patient was free of any complaints or complications.

## 1. Introduction

The foot’s extensor tendons are susceptible to laceration (e.g., with a sharp object penetration) because of their superficial course [1]. Acute complete rupture of these tendons usually results in a high-stepping, drop-foot gait and weakness of the ankle dorsiflexion and toe extension [2]. While end-to-end repair is performed in the majority of cases, delayed reconstruction is usually applied when potential clinical improvement outweighs the risks of surgery [3]. Here, for prompt management, ultrasound (US) or magnetic resonance imaging (MRI) is the mainstay in the diagnostic algorithm.

This report presents a unique case whereby US imaging played a paramount role in the diagnosis and surgical management of a previous nonanatomic repair of the ankle extensor tendons after a penetrating injury one year prior. Only a few observations of combined traumatic injuries of ankle extensor tendons have been reported previously. To the best of our knowledge, this is the first case of nonanatomic repair of combined traumatic injury of all three ankle extensor tendons.

## 2. Case Presentation

One year after a repair of the left ankle extensor tendons, in June 2020, a physically active 46-year-old female (otherwise a healthy retail cashier) was seen for persisting pain, tenderness, and fatigue along her left foot’s dorsal side since the surgery. She declared that the complaints got worse during hiking activities and that she also experienced a partial foot drop when walking barefoot. She denied any loss of sensation, dysesthesia, or paresthesia in her ankle/foot region. On detailed questioning, she reported that the surgery, as mentioned earlier, had occurred under emergency conditions following traumatic laceration of her left ankle extensor tendons (due to the sharp snowboard edge when another person passed over her foot).

**Figure 1:**
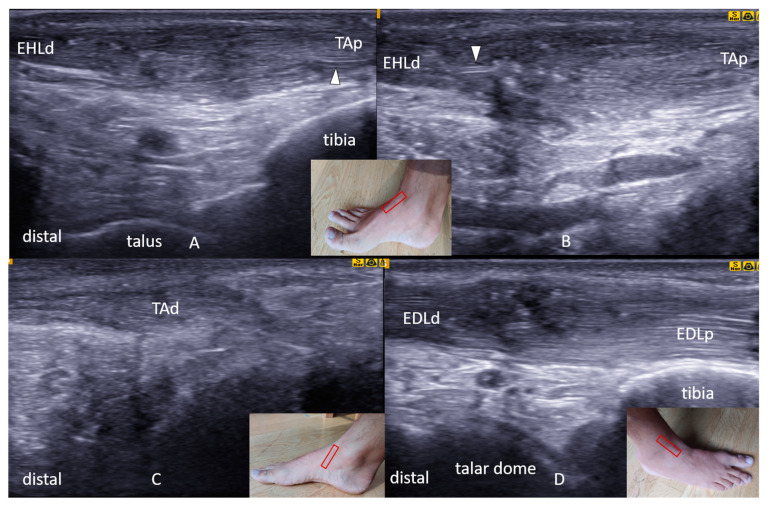
(**A**,**B**) Longitudinal ultrasound imaging of the dorsal aspect of the left ankle showed that the EHLd has clear continuity with the TAp. The junction is hypoechoic, thickened, and disorganized with visible suture material (white arrowheads). (**C**) Longitudinal ultrasound imaging of the anteromedial aspect of the ankle showed that the TAd stump ends blindly, attached to the subcutaneous tissue proximally. (**D**) The extensor digitorum longus tendon evaluation showed a normal anatomic course of the proximal and distal parts of the sutured tendons. The insets show the transducer positions (red rectangles) during scanning. EHLd, extensor hallucis longus distal end; TAp, tibialis anterior proximal end; TAd, tibialis anterior distal end; EDLp, extensor digitorum longus proximal end; EDLd, extensor digitorum longus distal end; EHLp, extensor hallucis longus proximal end; TA, tibialis anterior tendon; EHL, extensor hallucis longus.

Her current physical examination revealed a “bowstringing sign” in the extensor hallucis longus tendon (EHL) during resisted extension of the great toe. Muscle strength was found to be decreased for great toe extension (3/5) and ankle dorsiflexion (4/5). The initial clinical suspicion was a failure of the tibialis anterior tendon suture or peritendinous adhesions after the repair, resulting in loss of gliding. Comprehensive US tracking (Samsung UGEO HM70A, Seoul, South Korea) of the ankle extensors (i.e., proximal–distal and distal–proximal) was performed. A dynamic examination was also performed to assess the tendon mobility or their interruption. All findings were confirmed using at least two perpendicular planes. US examination revealed nonanatomic repair of the EHL and tibialis anterior (TA) tendons. In particular, the TA tendon’s proximal end showed continuity with the distal end of the EHL, where the sutured junction showed severe swelling and fiber disorganization (Figure 1A,B). The distal part of the TA tendon seemed to end blindly, i.e., attached to the subcutaneous tissue (Figure 1C). The proximal part of the EHL showed retraction, atrophy, and attachment to the lateral side of TA fascia. The extensor digitorum longus (EDL) tendon showed a correct anatomic course of its (sutured) proximal and distal parts (Figure 1D).

**Figure 2:**
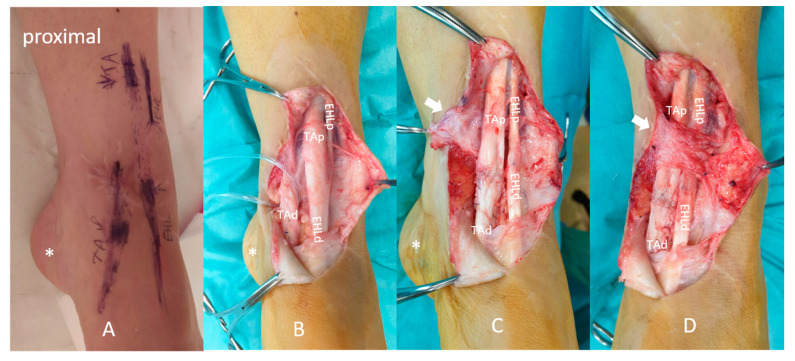
(**A**) The skin markings for the EHL and TA tendons. (**B**) Perioperative findings confirmed the non-anatomic repair of the EHL and TA tendons, i.e., the TAp tendon showed continuity with the EHLd. The sutured junction of the tendons showed severe swelling, indicating inappropriate healing. The TAd ended blindly, attached to the subcutaneous tissue. The EHLp showed retraction, atrophy, and attachment to the TA fascia (lateral side). (**C**,**D**) During surgery, end-to-end anatomic reconstruction was performed for both the EHL and TA tendons. EHLd, extensor hallucis longus distal end; EHLp, extensor hallucis longus proximal end; TAp, tibialis anterior proximal end; TAd, tibialis anterior distal end; asterisk (*), heel; white arrow, extensor retinaculum.

The physician who performed the US examination marked the precise localization of the EHL and TA tendons on the skin (Figure 2A) and referred the patient to surgery. As she consented to undergo surgery, “US-guided” repeat tendon reconstruction was performed, whereby all of the above-quoted findings were confirmed (Figure 2) and corrected with end-to-end sutures (Figure 2C,D).

A rehabilitation program was prescribed to restore the normal ankle joint range of motion and muscle strength, as well as to prevent peritendinous adhesions. It consisted of the following phases: Immobilization, early passive mobilization, and early active mobilization. The initial period comprised immobilization in a short-leg splint (with the ankle in the neutral position) and restriction of weight bearing for three weeks, following by another three weeks in a walking cast. Three weeks postoperatively, a rehabilitation program was initiated, with two to three therapy sessions per week. The initial physical therapy comprised progressive passive extension and relaxation of the toes and the ankle. Subsequently, in a stepwise fashion, the patient progressed to active flexion and extension—with a gradual increase in effort. Full weight bearing was gradually allowed between the postoperative third and six weeks. Stepwise/progressive return to hiking activities was allowed three months after the surgery. At the third postoperative month follow-up, the patient was free of any complaints or complications. Of note, the “bowstringing phenomenon,” as well as the muscle weakness, also disappeared. At this time, the follow-up US examination did not reveal any tendon suture failure or other serious abnormalities (apart from persistent tendinous thickening at the suture site).

## 3. Discussion

In this report, we presented a patient with persistent pain, tenderness, and fatigue due to the nonanatomic repair of the EHL and TA tendons, diagnosed with US examination. Apart from possible complications related to the anesthesia and the surgery itself (e.g., wound infection, pain, bleeding, and iatrogenic injury), the risk of repeat surgery was the failure to perform end-to-end tendon sutures. In this case, allografting or tendon transfer might have actually been necessary. Although tendon repair failure was a possible late complication in our patient, full recovery with no functional limitations was unlikely in our patient without surgery. As the patient was physically active before the injury and was motivated to recover with no substantial limitations to perform recreational sports (hiking, in particular), she opted for the surgical procedure. Eventually, with comprehensive physiotherapy, the outcome was satisfactory.

In the hitherto literature, most case reports/series of acute ankle and foot tendon injuries reported primary repair without any substantial delay. Those cases were usually isolated tendon injuries. Floyd et al. [4] reported a large series of 80 patients with open injuries of various tendons around the foot and ankle (excluding the Achilles tendon). Among the extensor tendons injured, EHL laceration was observed in 13 cases, EDL in eight cases, and TA in four cases. Five of the 80 patients had secondary repair (e.g., due to delayed diagnosis or wound infection). 

Isolated or associated TA lacerations can either occur after a traumatic or as a spontaneous injury and are more common in males in the sixth decade [5]. As TA provides approximately 80% of the ankle’s dorsiflexion [6], its preservation is vital for maintaining the normal function of the ankle/foot. After acute rupture, the proximal end of the tendon is usually retracted 3 cm or more, whereas the distal end is commonly found in the (skin) laceration site. Despite being erroneous, as the proximal end of the TA tendon was sutured to the distal EHL tendon, there was not significant retraction and end-to-end repair could have been performed for TA. Delayed tendon repairs (later than three months after complete transection) are generally believed to be associated with less favorable outcomes [3]. Regarding TA tendon rupture, in their systematic review with a meta-analysis, Tickner et al. [5] reported seven months as the average delay in diagnosis. Additionally, due to tendon retraction, the need for (allo) grafting has even been reported in late cases of TA tendon rupture [7]. Of note, some authors do not recommend using EHL as an autograft because of the likely loss of ankle dorsiflexion strength [5].

A rare case of combined traumatic laceration of TA and EHL was reported by Franck et al. [8], where the correct diagnosis was made upon physical examination and magnetic resonance imaging, with a two-week delay. During surgery, the authors found the tendons significantly retracted, and they performed a functional nonanatomic reconstruction with tendon transfers.

An injury to the EHL tendon usually results in loss/weakness of the great toe extension. While acute laceration of the tendon usually requires repair to prevent deformity, in isolated cases (and/or when surgery is not possible), wearing a shoe might often be sufficient to maintain the functional capacity of the subject [9]. Similar to the TA, EHL tendon lacerations need to be repaired in the first three months of injury [3]. Kurashige et al. [10] reported a case of chronic EHL tendon rupture diagnosed with magnetic resonance imaging. In their work, the authors described successful grafting with the use of an accessory extensor hallucis capsularis tendon. Again, as the proximal stump of EHL in our patient remained attached (side-to-side) to the proximal TA tendon, there was no retraction precluding end-to-end repair. Furthermore, the existing nonanatomic suture between the proximal stump of TA and the distal stump of EHL tendons may explain the residual great toe extension strength (3/5, as measured by manual muscle testing). In addition, we speculate that extramuscular myofascial force transmission (through connective tissue linkages) also played at least a partial role in the great toe function preservation. As regards cases of chronic EDL rupture, some authors believe that the delay in recognition of EDL does not require repair if the extensor digitorum brevis muscle remains intact [3].

Although there are other methods to image the common injuries of the ankle/foot (e.g., computed tomography and magnetic resonance imaging), US is the one that presents additional benefits [11,12]. Indeed, it provides a comprehensive and real-time static/dynamic examination of the joints and nearby tendons, nerves, and ligaments.

## 4. Conclusions

Herein, we presented a case of delayed diagnosis of a non-anatomic repair of the EHL and TA tendons after a penetrating injury. It is noteworthy that US examination provided a better understanding of the local “abnormal” anatomy and promptly navigated the repeat surgery. As such, we strongly advocate the use of US in presurgical planning.

## Data Availability

No datasets were generated or analyzed.
